# L-Arginine and Nitric Oxide in Vascular Regulation—Experimental Findings in the Context of Blood Donation

**DOI:** 10.3390/nu17040665

**Published:** 2025-02-13

**Authors:** Natalia Kurhaluk, Halina Tkaczenko

**Affiliations:** Institute of Biology, Pomeranian University in Słupsk, Arciszewski St. 22b, 76-200 Słupsk, Poland; halina.tkaczenko@upsl.edu.pl

**Keywords:** vascular health, endothelial function, oxidative stress, blood donation, vasodilation, personalised nutrition, dietary interventions

## Abstract

This narrative review provides an analysis of the role of nitric oxide (NO) and its precursors, particularly L-arginine, in vascular regulation and health, with an emphasis on findings from our experimental research in animal models. NO serves as a critical mediator of vascular function, contributing to vasodilation, the regulation of blood flow, and the prevention of thrombosis. As a primary precursor of NO, L-arginine is essential for maintaining endothelial integrity, modulating mitochondrial function, and reducing oxidative damage. This review synthesises the data and contextualises these findings within the physiological challenges faced by blood donors, such as repeated blood donation and associated oxidative stress. It examines the effects of L-arginine supplementation on mitochondrial respiration, lipid peroxidation, and microsomal oxidation in different conditions, including differences in age, gender, and dietary interventions. The mechanisms by which L-arginine enhances NO production, improves vascular elasticity, and alleviates endothelial dysfunction caused by reduced NO bioavailability are also investigated. By integrating experimental findings with insights from the existing literature, this review provides a perspective on the potential of L-arginine supplementation to address the specific physiological needs of blood donors. It highlights the importance of personalised nutritional approaches in enhancing donor recovery and vascular resilience. In addition, this review assesses the wider implications of L-arginine supplementation in mitigating oxidative stress and preserving vascular function. The interplay between NO bioavailability, dietary factors, and physiological adaptation in blood donors is highlighted, along with the identification of current knowledge gaps and recommendations for future research. By presenting both original experimental evidence and a critical synthesis of the literature, this article highlights the therapeutic potential of NO precursors, particularly L-arginine, in promoting vascular health in the context of blood donation.

## 1. Introduction

L-arginine, classified as a semi-essential amino acid, plays a central role in numerous physiological processes, most notably as a precursor for nitric oxide (NO) synthesis [1]. NO is produced enzymatically from L-arginine by nitric oxide synthases (NOS) and exerts far-reaching effects on cellular signalling and general health [2]. As a critical regulator of homeostasis, NO contributes significantly to the maintenance of vascular tone and modulation of immune responses, highlighting its essential role in diverse biological systems [3].

Nitric oxide plays a particularly important role in the cardiovascular system. By facilitating vasodilation, it promotes efficient blood flow and oxygen delivery to tissues, inhibits platelet aggregation, and modulates vascular inflammation [4]. Beyond its cardiovascular functions, NO contributes to immune defence, neurotransmission, and mitochondrial regulation, highlighting its multifaceted biological importance [5]. Under physiological stress, the interplay between L-arginine and NO becomes particularly critical for maintaining vascular health and resilience [6].

Blood donation is a unique physiological challenge that triggers adaptive responses to compensate for the loss of blood volume and cellular components [7]. Despite being a safe and well-regulated procedure, it induces transient haemodynamic changes, oxidative stress, and a transient decrease in haemoglobin levels [8,9]. These physiological perturbations highlight the importance of effective recovery mechanisms to restore homeostasis and ensure donor well-being. In this context, L-arginine and NO are emerging as key contributors to recovery due to their roles in improving endothelial function, attenuating oxidative stress, and enhancing vascular elasticity [10]. Understanding these mechanisms not only illustrates the remarkable adaptability of the body but also points to innovative strategies for optimising donor health through personalised dietary and lifestyle interventions.

The intersection of L-arginine and NO with blood donation represents a timely and relevant area of investigation in contemporary health and medicine. Research into the molecular mechanisms underlying NO production and its impact on vascular health extends beyond the realm of blood donation, revealing broader implications for cardiovascular health, immune function, and metabolic regulation. These findings are particularly relevant to conditions such as hypertension [11], diabetes [12,13], and chronic inflammatory diseases [14]. Therefore, an investigation of the role of L-arginine supplementation and NO pathways in blood donors offers valuable insights with potential therapeutic applications for the general population.

Recovery after blood donation is a critical physiological process that requires adaptive responses to compensate for the temporary loss of blood volume and red blood cells [15]. Diet plays a pivotal role in this recovery, as specific nutrients are required to restore homeostasis and replenish lost components [16,17]. For example, iron, found in red meat, spinach, and legumes, is essential for the regeneration of haemoglobin and restoration of oxygen-carrying capacity [18], while vitamin C enhances iron absorption from plant sources [19]. L-arginine contributes to recovery by facilitating the production of NO, which improves vascular function, reduces oxidative stress, and supports endothelial health [10]. Adequate hydration also aids recovery by rapidly restoring the blood volume [17]. A nutrient-rich, well-balanced diet not only accelerates recovery but also improves donor well-being by minimising complications, such as fatigue, dizziness, and prolonged recovery times [20,21].

This integrative approach links the physiological response to blood donation to broader health challenges, including endothelial dysfunction, oxidative stress, and ageing. In the context of the ageing population and the increasing prevalence of chronic diseases, optimising vascular health and reducing oxidative damage are increasingly important priorities [22]. L-arginine supplementation is emerging as a promising strategy to improve cardiovascular function, reduce oxidative stress, and promote overall well-being. These benefits extend beyond blood donors to such populations as cardiovascular patients, surgical candidates, and athletes seeking enhanced recovery [23,24]. In addition, this research is consistent with the emerging trends in personalised medicine, which emphasise the importance of tailoring interventions to individual factors, such as age, gender, genetics, and diet. Precision-based approaches are gaining traction for their potential to provide targeted and effective health management [25]. Understanding the multifaceted effects of L-arginine in different populations and contexts provides groundbreaking insights that enable the development of tailored strategies to meet individual health needs.

The aim of this review is to examine the role of L-arginine and NO in the context of blood donation, focusing on the molecular mechanisms of NO synthesis, its impact on vascular health, and its contribution to post-donation recovery. This review evaluates dietary and endogenous sources of L-arginine and highlights its physiological importance in supporting endothelial function, mitigating oxidative stress, and enhancing donor recovery. In addition, this review explores the therapeutic potential of L-arginine supplementation to improve donor health, highlighting the critical role of nutrition in optimising post-donation recovery outcomes.

## 2. Methodology

This article is based on data collected through an extensive review of primary and secondary literature, focusing on experimental studies, original research, clinical trials, and reviews published within the last two decades, using a range of databases including PubMed, Web of Science, Scopus, Cochrane Library, Embase, and Google Scholar. The search strategy used a combination of keywords and MeSH terms, such as ‘L-arginine’, ‘nitric oxide’, ‘vascular health’, ‘endothelial function’, ‘oxidative stress’, ‘blood donation’, ‘vasodilation’, ‘nutrition’, and ‘dietary interventions’, together with the use of Boolean operators (AND, OR) to refine the results. Inclusion criteria were peer-reviewed studies focusing on the effects of L-arginine on nitric oxide production in relation to its effects on vascular health. Studies were selected if they were clinical trials, observational studies, or systematic reviews directly related to endothelial function and vascular relaxation. The review highlights key topics such as the molecular pathways of nitric oxide (NO) synthesis, the role of dietary sources in L-arginine metabolism, and the effects of blood donation on vascular function. In addition, data from animal models, human trials, and mechanistic studies were integrated to provide a comprehensive perspective on the role of L-arginine and NO in supporting recovery after blood donation. Studies were considered ineligible for inclusion in this review if they met any of the following criteria: (1) non-peer-reviewed publications, including opinion pieces, commentaries, editorials, conference abstracts, case reports, and non-systematic narrative reviews; (2) research that did not include experimental, observational, clinical trials, or systematic reviews, such as theoretical models without empirical validation; (3) studies that did not include a comprehensive methodological description, robust statistical analyses, or reported inconclusive results without adequate experimental support.

### 2.1. L-Arginine as a Critical Amino Acid for NO Synthesis

**Figure 1:**
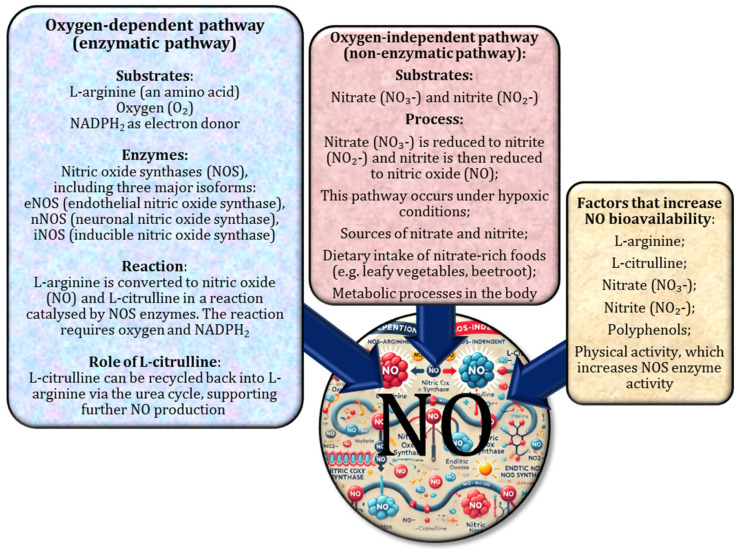
Pathways of nitric oxide (NO) biosynthesis. This diagram shows the two pathways of NO production. The oxygen-dependent pathway involves the conversion of L-arginine to NO and L-citrulline by nitric oxide synthase (NOS) enzymes with oxygen and NADPH_2_. The oxygen-independent pathway reduces nitrate (NO_3_^−^) to nitrite (NO_2_^−^) and then to NO under hypoxic or acidic conditions. Factors such as L-arginine, L-citrulline, nitrate, nitrite, and polyphenols increase the bioavailability of NO.

Nitric oxide is produced by two different pathways: one dependent on nitric oxide synthase (NOS) and the other independent of NOS [1,2]. In the NOS-dependent pathway, NO is synthesised from L-arginine and oxygen in a reaction catalysed by NOS enzymes, such as endothelial nitric oxide synthase (eNOS). In addition, L-citrulline, an α-amino acid, contributes to NO production in this pathway by being converted to L-arginine, which further supports NO synthesis. In the NOS-independent pathway, nitrate (NO_3_^−^) is converted to nitrite (NO_2_^−^) and subsequently to NO. It has been suggested that NO availability can be increased by increasing the levels of NO_3_^−^, NO_2_^−^, L-arginine, L-citrulline, or polyphenols [3,4,5]. Changes in NO bioavailability are commonly assessed by measuring plasma or urinary NO_3_^−^ and NO_2_^−^, which are the end products of endogenous NO synthesis. In addition, NO bioavailability can be inferred from assessments of vascular function, such as flow-mediated dilation [26]. The pathways of NO biosynthesis are shown in Figure 1.

L-arginine, a vital amino acid that is central to the production of NO, is metabolised within endothelial cells by the enzyme nitric oxide synthase [1]. This process results in the production of NO, which plays a key role in dilating blood vessels, maintaining vascular tone, and facilitating proper blood flow [3,27]. By promoting vasodilation, NO helps to lower blood pressure and improve oxygen delivery to tissues, supporting overall cardiovascular health [4,28]. In addition, the anti-inflammatory and anti-thrombotic properties of NO protect the endothelium and reduce the risk of clot formation [29,30]. Given its importance, L-arginine is essential not only for maintaining healthy circulation but also for managing physiological stress, such as that experienced during blood donation or physical exertion [1,24].

**Figure 2:**
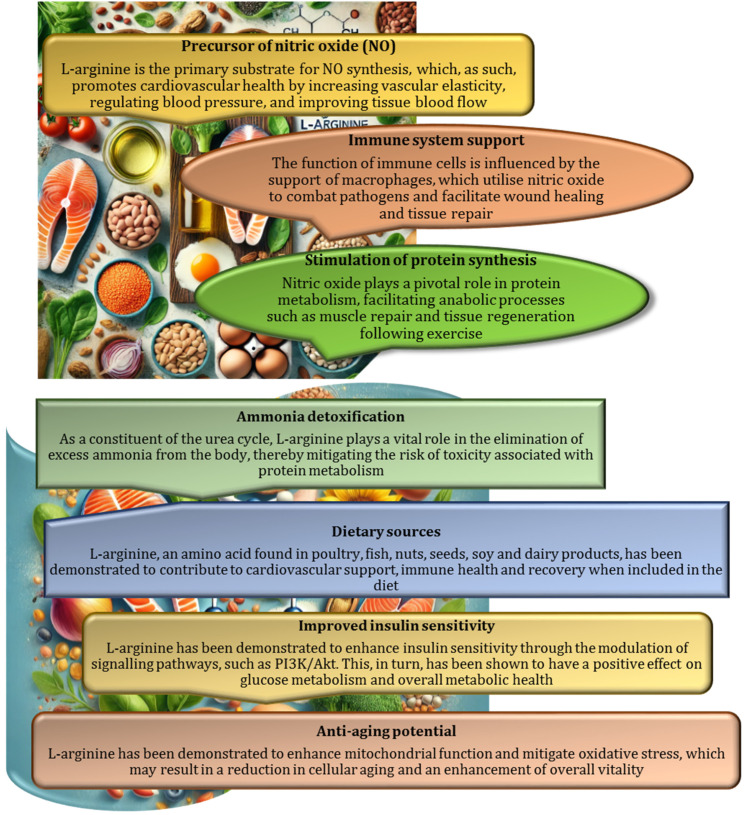
Key properties of L-arginine in nutrition and health. L-arginine is a multifunctional amino acid that is essential for nitric oxide (NO) production and overall health. It has dietary and therapeutic applications in sports nutrition, cardiovascular health, and metabolic support.

Dietary intake is the most accessible way to maintain adequate levels of L-arginine [31,32,33], as it has a direct effect on nitric oxide production and vascular health. Nuts, seeds, meat, fish, dairy products, and plant proteins are rich sources of L-arginine. Among plant sources, almonds, walnuts, sunflower seeds, legumes, soybeans, and lentils are excellent sources that provide L-arginine along with other nutrients, such as healthy fats, proteins, and essential vitamins [34,35]. Foods of animal origin, such as beef, poultry, fish (e.g., salmon), and dairy products, are particularly rich in L-arginine, which contributes significantly to endothelial function and NO synthesis [36]. For subjects on a plant-based diet, legumes and soy products offer effective alternatives to ensure adequate intake of this amino acid. Incorporating a balanced diet that includes both animal and plant sources of L-arginine can significantly increase the body’s ability to produce nitric oxide, thereby promoting vascular health and overall well-being [35,37]. The nutritional and health benefits of L-arginine are summarised in Figure 2.

Over the last few decades, extensive research has established that NO synthesis is primarily catalysed by the NOS family of enzymes, which includes endothelial NOS (eNOS), neuronal NOS (nNOS), and inducible NOS (iNOS). While these enzymes share a common catalytic mechanism, they are expressed in different tissues and regulated by different factors [38]. eNOS is predominantly found in endothelial cells, where it regulates vascular tone and blood flow by producing NO in response to such stimuli as shear stress and acetylcholine [3,39]. nNOS is predominantly expressed in neurons and plays an important role in neurotransmission [40], while iNOS is induced during immune activation and produces high levels of NO as part of the body’s defence against pathogens or inflammation [41].

Building on fundamental studies, recent research has focused on the enzymatic conversion of L-arginine to NO, a process mediated by NOS enzymes [38,42]. This involves the oxidation of L-arginine, aided by molecular oxygen and cofactors, resulting in the production of NO and citrulline. The reaction takes place in two steps: first, L-arginine is hydroxylated to form N-hydroxy-L-arginine, which is then further oxidised to form NO and citrulline. The efficiency of this process is highly dependent on the availability of such cofactors as tetrahydrobiopterin (BH4). BH4 is essential for the functionality of NOS, stabilising the enzyme and facilitating the electron transfer required for catalysis [43,44]. Insufficient levels of BH4 can impair NO production, leading to endothelial dysfunction and associated cardiovascular complications [45].

Recent studies have highlighted the significant impact of several factors on the efficiency and rate of NO synthesis, in particular, the availability of essential cofactors and oxygen levels [46,47]. Tetrahydrobiopterin is a critical cofactor for nitric oxide synthase activity, ensuring that the enzyme remains active and is able to produce NO [48]. A deficiency of BH4 can lead to the uncoupling of NOS, resulting in the production of superoxide instead of NO, contributing to oxidative stress and endothelial dysfunction [49]. Oxygen levels are also critical as NOS enzymes require oxygen to oxidise L-arginine [38]. Research has shown that hypoxia or low-oxygen conditions can significantly reduce NO synthesis, highlighting the need for an adequate supply of both oxygen and cofactors to maintain optimal NO production and vascular health [50].

The regulation of NOS enzymes and NO synthesis is a highly complex process influenced by multiple factors, including gene expression, post-translational modifications, and external stimuli. For example, eNOS activity is modulated by shear stress, intracellular calcium levels, and phosphorylation by specific kinases [51]. In contrast, iNOS expression is primarily driven by inflammatory cytokines during immune responses [52]. Similarly, nNOS activity is regulated by neuronal signalling and fluctuations in intracellular calcium levels [53]. These finely tuned regulatory mechanisms ensure that NO production is adapted to physiological needs [47]. Understanding these molecular pathways is crucial for the development of therapeutic strategies aimed at improving vascular health, modulating immune responses, and supporting neurotransmission.

### 2.2. Metabolic Pathways of NO Synthesis in the Body

Nitric oxide is synthesised via two different pathways: a nitric oxide synthase (NOS)-dependent pathway and a NOS-independent pathway. In the NOS-dependent mechanism, NO is produced from L-arginine and oxygen by enzymatic reactions catalysed by NOS isoforms, such as endothelial nitric oxide synthase (eNOS) [54,55,56]. L-citrulline, i.e., an α-amino acid, further supports NO synthesis in this pathway by being metabolised to L-arginine, thereby maintaining substrate availability. In contrast, the NOS-independent pathway involves the sequential reduction of nitrate (NO_3_^–^) to nitrite (NO_2_^–^) and subsequently to NO. Increased NO bioavailability can be achieved by increasing the levels of NO_3_^–^, NO_2_^–^, L-arginine, L-citrulline, or polyphenols [57]. Common methods for assessing NO bioavailability include the measurement of plasma or urinary levels of NO_3_^–^ and NO_2_^–^—the end products of endogenous NO synthesis—and functional assessments of vascular health, such as flow-mediated dilation [26].

L-arginine, which is a semi-essential amino acid central to NO production, is metabolised in endothelial cells by NOS activity [1]. This enzymatic reaction facilitates the generation of NO, a molecule critical for vasodilation, the maintenance of vascular tone, and optimal blood flow [3,27]. NO-mediated vasodilation lowers blood pressure and increases oxygen delivery to tissues, contributing to cardiovascular health [4,28]. In addition, NO has anti-inflammatory and anti-thrombotic properties that protect endothelial integrity and reduce thrombus formation [29,30]. The importance of L-arginine extends to contexts of physiological stress, such as blood donation or strenuous physical activity, highlighting its role in circulatory homeostasis [1,24].

Although L-arginine is widely recognised as the primary precursor for NO synthesis, recent evidence suggests that alternative mechanisms also contribute to NO production. In addition to L-arginine, dietary nitrates and nitrites, endogenous nitrate reduction pathways, mitochondrial NO production, and the reduction of nitrogen metabolites to NO have been identified as additional sources of NO [58,59]. Recent studies have suggested a potential link between dietary nitrates and nitrites [60,61]. Nitrates, which are commonly found in vegetables such as beetroot, lettuce, and spinach, can be reduced in the body to nitrites, which are subsequently converted to NO [62]. This mechanism is particularly important for consumers of nitrate-rich vegetables, as it provides an alternative source of NO that does not rely on L-arginine. Research has highlighted the importance of endogenous nitrate reduction processes, whereby dietary nitrates are absorbed into the bloodstream and stored in blood vessel walls. Through the action of enzymes such as nitrate reductase, these nitrates are reduced to nitrites, which are further converted to NO [63]. This process is essential for maintaining vascular health, especially during oxidative stress or when L-arginine availability is compromised [64].

Recent evidence also highlights mitochondrial sources of NO, suggesting that mitochondria play a role in its production [65]. Mitochondrial nitric oxide synthase (mtNOS) generates NO within cells, particularly in energy-demanding tissues, such as muscle [66]. NO produced in the mitochondria is involved in the regulation of cellular metabolism and provides protection against oxidative stress [67]. In addition, the reduction of nitrogen metabolites to NO is an important process in certain pathological states. Although this pathway is not fully understood, it is thought to occur in immune cells and tissues under high oxidative stress, where NO is synthesised from alternative nitrogen precursors [47,68]. These findings highlight that while L-arginine is the primary source of NO, alternative mechanisms provide complementary pathways, especially when L-arginine synthesis is impaired, e.g., in inflammatory conditions or hypertension [69,70].

### 2.3. Vascular Tone and Blood Pressure as Major Functions of NO

The primary role of NO is to regulate vascular tone and blood pressure [71]. NO is synthesised in the endothelium by eNOS from L-arginine. After production, NO diffuses into vascular smooth muscle cells, where it activates guanylate cyclase. This enzyme increases the levels of cyclic guanosine monophosphate (cGMP), leading to smooth muscle cell relaxation and subsequent vasodilation. This mechanism is essential for the regulation of vascular tone and blood pressure, ensuring adequate blood flow and oxygen delivery to tissues [72]. Dysfunction or impaired NO production can lead to hypertension and other cardiovascular diseases [73]. In addition, NO plays a protective role in maintaining endothelial health and preventing atherosclerosis by preserving endothelial homeostasis [39,74]. It inhibits leukocyte adhesion, reduces the expression of pro-inflammatory cytokines, and prevents smooth muscle cell proliferation, all of which are involved in plaque formation [38,75]. In addition, NO reduces oxidative stress in the endothelium by scavenging free radicals, thereby maintaining vascular integrity. This protective function highlights the role of NO in attenuating the early stages of atherosclerosis and supporting overall vascular health [76].

Another important function of NO is its antiplatelet and antithrombotic effects [77,78]. NO has significant antithrombotic properties, as it inhibits platelet aggregation and adhesion to the vessel wall. This is achieved by its ability to increase platelet cyclic guanosine monophosphate (cGMP) levels, thereby reducing platelet activation [79]. By limiting excessive platelet activity, NO contributes to a reduced risk of thrombosis, a key factor in acute cardiovascular events, such as myocardial infarction and stroke. These antiplatelet effects position NO as a natural defence against clot-related complications in the circulatory system [80]. In summary, the multiple roles of NO are integral to cardiovascular health, and its dysregulation is strongly associated with the development of various vascular diseases [81].

Nitric oxide exerts its physiological effects primarily by activating soluble guanylyl cyclase (sGC) in target cells. By binding to the haem group of sGC, NO induces a conformational change that significantly enhances the activity of the enzyme. This activation catalyses the conversion of guanosine triphosphate (GTP) into cyclic guanosine monophosphate (cGMP), an important second messenger involved in a variety of cellular processes. Increased levels of cGMP activate protein kinase G (PKG), which phosphorylates specific target proteins, leading to a reduction in intracellular calcium levels. This decrease in calcium facilitates smooth muscle relaxation, promoting vasodilation and improving blood flow regulation [82,83].

**Figure 3:**
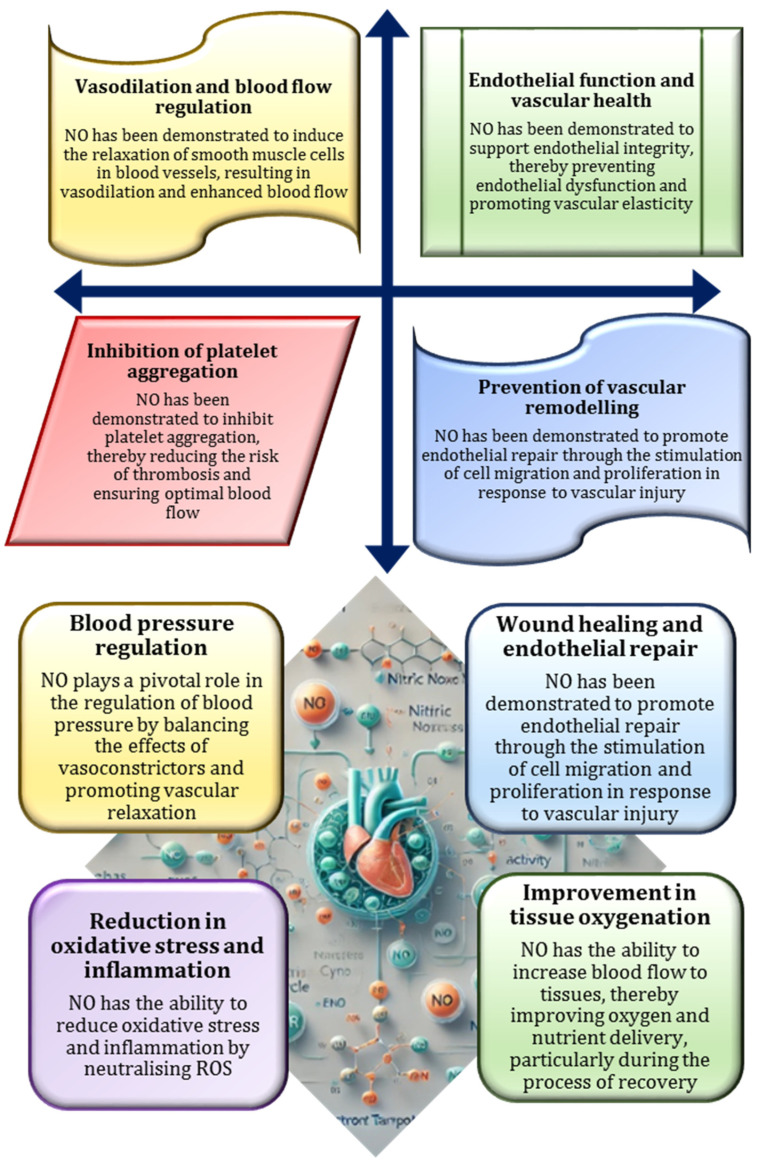
Role of nitric oxide in vascular health and function. Nitric oxide plays a pivotal role in the maintenance of healthy blood vessels and overall vascular function, with critical functions including vasodilation, blood flow regulation, thrombosis prevention, and endothelial health. In addition, NO has been shown to have multiple actions in the prevention of cardiovascular disease and in supporting recovery processes during blood donation. ROS—reactive oxygen species.

Recent studies have highlighted the dynamic interaction between NO and reactive oxygen species (ROS), which significantly affects NO bioavailability and cellular functions [84]. In an oxidative environment, NO reacts with superoxide anions (O_2_^−^) to form peroxynitrite (ONOO^−^), a highly reactive compound capable of causing oxidative damage to lipids, proteins, and DNA. This reaction reduces the availability of NO, impairing its vasodilatory and protective roles [85]. Conversely, in a balanced redox environment, NO helps to alleviate oxidative stress by scavenging free radicals [76]. The delicate balance between NO and ROS is critical in conditions such as atherosclerosis, where increased oxidative stress depletes NO levels, disrupts endothelial function, and promotes vascular injury [86]. The critical role of NO in vascular health and function is illustrated in Figure 3.

The molecular mechanisms underlying the action of nitric oxide underscore its central role in vascular health and its potential as a therapeutic target for cardiovascular and oxidative stress-related diseases, particularly those associated with redox balance and antioxidant defences [87]. NO is critical for maintaining cellular redox homeostasis by modulating antioxidant systems. It increases the expression of antioxidant enzymes, such as superoxide dismutase (SOD) and catalase, which reduce the accumulation of ROS [88]. In addition, NO regulates the levels of glutathione, a key antioxidant molecule that protects cells from oxidative damage [89].

By maintaining redox balance, NO prevents the activation of pro-inflammatory pathways and inhibits cellular apoptosis, thereby supporting both vascular and systemic health [90]. These mechanisms highlight the dual role of NO: its protective effects against oxidative damage depend on its concentration and the prevailing cellular redox state, while its availability and function can also be modulated by the oxidative environment. This duality highlights the delicate balance that NO maintains in promoting health while being vulnerable to disruption by oxidative stress [91].

### 2.4. NO Bioavailability and Diet

Numerous studies have highlighted the important role of diet in improving nitric oxide bioavailability, as NO production is closely linked to specific dietary components, in particular, dietary nitrates and amino acids, such as L-arginine and L-citrulline [26,92]. These substances act as important precursors for NO synthesis via endothelial nitric oxide synthase. Research suggests that the consumption of nitrate-rich foods, such as leafy greens, beets, and citrus fruits, significantly increases NO production, thereby promoting vasodilation and improving circulation [93,94]. Consequently, maintaining optimal NO levels through appropriate nutrition is crucial for blood donors, as it supports vascular function, reduces post-donation fatigue, and facilitates recovery [95]. A deficiency of these essential nutrients can impair NO synthesis, negatively impacting vascular health and delaying recovery after donation.

The interaction between NO, oxidative stress, and donor health is well established in the existing research. Blood donation induces oxidative stress due to transient hypovolemia and associated physiological adaptations [8,96,97]. During this process, NO interacts with ROS and affects the redox balance in the body [98]. While NO can neutralise free radicals to reduce oxidative stress, an excess of ROS can lead to the formation of peroxynitrite, which depletes NO levels and impairs endothelial function [85]. Studies suggest that dietary antioxidants, including vitamins C and E and polyphenols, increase NO stability and counteract oxidative damage [99,100,101,102,103]. Blood donors are encouraged to consume an antioxidant-rich diet to protect against oxidative stress, promote vascular health, and support faster physiological recovery [17].

**Figure 4:**
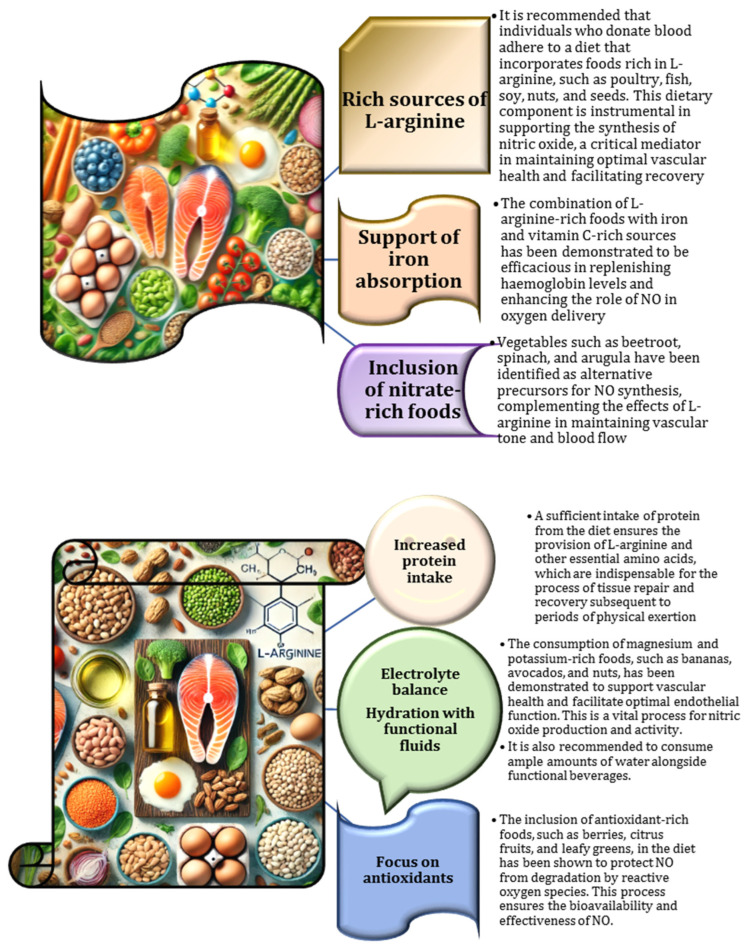
Relationship between the blood donor’s diet and the presence of L-arginine and nitric oxide. A diet for blood donors that is well-structured and emphasises L-arginine and NO-related nutrients accelerates recovery and improves overall vascular health, thereby facilitating regular donations.

Recent research has emphasised the importance of dietary strategies for blood donors aimed at optimising NO bioavailability, highlighting the importance of a balanced nutrient intake to support endothelial health and reduce oxidative stress [104,105]. The key recommendations include the inclusion of nitrate-rich vegetables, amino acids (from sources such as nuts, seeds, or supplements), and antioxidants to stabilise NO levels [64]. These dietary interventions not only facilitate donor recovery but also help prevent such complications as hypoperfusion and platelet aggregation by exploiting the vasodilatory and antiplatelet properties of NO [80,106]. Furthermore, customising dietary guidelines based on individual factors, such as gender and metabolic requirements, may further enhance NO functionality and promote a safer and more effective blood donation process [107]. The specific dietary considerations related to L-arginine and NO for blood donors are outlined in Figure 4.

### 2.5. Risks and Potential Side Effects of NO Dysregulation

Recent research suggests that excessive NO production, particularly through the upregulation of inducible nitric oxide synthase (iNOS), can contribute to oxidative stress and tissue damage via the formation of reactive nitrogen species, such as peroxynitrite. This reactive molecule disrupts cellular integrity by damaging lipids, proteins, and nucleic acids [108]. In blood donors, oxidative stress associated with NO overproduction may be exacerbated by iron overload resulting from iron supplementation. Elevated iron levels may catalyse the Fenton reaction, generating additional ROS and exacerbating oxidative damage, thereby compromising endothelial and vascular health [109].

Extensive research conducted over the years has highlighted the critical role of iron as a micronutrient essential for several physiological processes, particularly the synthesis of haemoglobin and myoglobin, which are essential for oxygen transport and storage [110,111]. Haemoglobin in red blood cells facilitates oxygen transport from the lungs to the tissues, while myoglobin in muscle stores oxygen. Iron is also a critical component of enzymes involved in metabolic pathways, including cellular respiration and energy conversion [18,110].

Dietary iron comes in two main forms: haem iron and non-haem iron. Haem iron, found mainly in animal-based foods, such as meat, poultry, and fish, is highly bioavailable and easily absorbed by the body. In contrast, non-haem iron, which makes up the majority of iron in plant-based sources, is less efficiently absorbed. Notably, over 95% of functional iron in the human body is in the haem form, highlighting its critical role in various physiological processes [112]. Iron homeostasis is primarily regulated by intestinal absorption and storage or release by the liver [113]. Transferrin, the major iron transport protein, delivers iron to tissues, while ferroportin and hepcidin are key regulators of iron balance [114]. Hepcidin, a hormone secreted by the liver, reduces intestinal iron absorption and limits iron release from macrophages and liver stores, thereby protecting against iron overload [115]. In contrast, ferroportin facilitates the transport of iron into the bloodstream [116]. A proper balance between these regulatory mechanisms is essential for maintaining health, as imbalances can lead to such conditions as anaemia or iron overload [117].

The importance of dietary iron for blood donors is well established [118]. Each blood donation results in a loss of approximately 200–250 mg of iron, and frequent donations can deplete iron stores, potentially leading to anaemia and delayed recovery [119,120]. To support recovery and maintain iron levels, blood donors are advised to consume iron-rich foods, including red meat, poultry, fish, and plant-based sources, such as lentils, beans, tofu, and leafy greens [17]. In addition, adequate vitamin C intake is essential to improve the absorption of non-haem iron, particularly for subjects on vegetarian or vegan diets [18]. Peppers, strawberries, and citrus fruits can significantly improve iron absorption [121].

**Figure 5:**
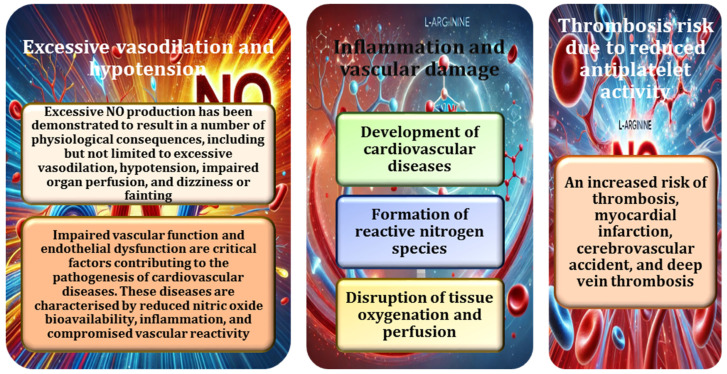
Fundamental mechanisms of risk and potential side effects associated with NO dysregulation. Chronic NO dysregulation has been demonstrated to exacerbate inflammation within the vasculature, thereby contributing to the development of cardiovascular disease, including atherosclerosis, by promoting the accumulation of inflammatory cells within blood vessel walls.

The mechanisms underlying nitric oxide dysregulation and the associated risks are illustrated in Figure 5. Chronic inflammation has been shown to play a central role in the development of vascular damage and the progression of cardiovascular disease [4,5,14]. In particular, dysregulation of NO production, whether excessive or defective, has been shown to exacerbate inflammation within the vasculature. Excessive NO production has been shown to lead to the formation of reactive nitrogen species (RNS), which have been shown to contribute to oxidative stress, further exacerbating inflammation and endothelial dysfunction [109]. This can lead to damage to the blood vessel walls and predispose to conditions such as atherosclerosis, where the vessel walls become thickened and hardened due to the accumulation of inflammatory cells and lipid deposits. Conversely, impaired NO production (e.g., due to endothelial dysfunction or inadequate availability of its precursor, L-arginine) impairs the vasodilatory function of NO, leading to reduced blood flow and increased susceptibility to vascular injury and chronic inflammation [86,87,88]. This dysfunction is a key factor in the development of cardiovascular disease as it compromises the ability of blood vessels to respond to changes in blood flow, leading to further damage and disease progression.

Both excessive and deficient levels of NO contribute significantly to vascular damage through their effects on inflammation, with the potential for long-term cardiovascular complications. In addition, L-arginine plays an essential role in NO production as a substrate for the enzyme nitric oxide synthase [4,5]. Supplementation with L-arginine can help restore NO balance and, in some cases, alleviate some of the inflammatory damage caused by NO dysregulation [6]. However, the effectiveness of L-arginine therapy depends on several factors, including the underlying condition causing the NO imbalance. Chronic dysregulation of NO has been shown to reduce its antiplatelet activity, thereby increasing the risk of thrombosis [80,106]. When NO production is impaired, the ability to prevent platelet aggregation is compromised, promoting the formation of blood clots, particularly in the vasculature. L-arginine, a precursor of NO, plays a crucial role in maintaining normal NO production; its deficiency can further exacerbate the reduction in antiplatelet function, thereby increasing the likelihood of thrombotic events such as heart attack and stroke [77,78].

Studies also highlight the potential toxic effects of iron in blood donors [122,123]. While iron is essential for several physiological functions, excessive accumulation thereof can pose significant health risks, especially in individuals who donate blood frequently. Iron overload can lead to tissue damage, particularly in vital organs, such as the liver, heart, and pancreas [124]. This condition results from iron accumulation, which facilitates the formation of ROS, leading to oxidative stress and cellular damage [109]. Regular blood donation and iron supplementation may exacerbate these risks, increasing the likelihood of cardiovascular diseases, e.g., atherosclerosis, and liver diseases, such as fatty liver disease or cirrhosis [125,126]. To reduce these risks, it is important to monitor iron levels in blood donors and, if necessary, adjust the dietary intake or provide supplementation of this element to prevent the adverse effects associated with iron overload [127,128].

### 2.6. Endothelial Dysfunction and the L-Arginine Paradox

The ’L-arginine paradox’ term refers to the counterintuitive observation that oral supplementation with L-arginine, i.e., a substrate for NOS-mediated NO synthesis, does not consistently lead to a marked increase in NO production [129]. This phenomenon can be attributed to the body’s limited capacity to utilise L-arginine for NO synthesis in specific conditions. A significant factor contributing to this paradox is the enzyme arginase, which competes with NOS for L-arginine. Elevated arginase activity has been demonstrated to reduce the pool of L-arginine available for NO synthesis, thereby limiting NO production despite higher L-arginine levels [129,130]. Furthermore, endothelial dysfunction and oxidative stress exacerbate the issue by impairing the conversion of L-arginine to NO. ROS have been shown to both degrade NO and inhibit NOS activity, thereby further compromising NO bioavailability [39,131].

A further dimension of the L-arginine paradox pertains to the role of endothelial cells and the mechanisms governing L-arginine transport across cell membranes. In conditions of oxidative stress or inflammation, the intracellular transport of L-arginine may be impaired, reducing its accessibility for NO synthesis. This is of particular relevance in cardiovascular diseases, where endothelial dysfunction is associated with diminished NO bioavailability [10,86]. In such contexts, L-arginine supplementation frequently fails to restore NO levels. Alternative strategies, such as enhancing endothelial function or upregulating eNOS activity, may be required to address the paradox effectively [132,133]. This highlights the complex interplay between L-arginine metabolism, NO synthesis, and vascular endothelial health [130,134].

Recent studies have increasingly focused on endothelial dysfunction in patients, which may stem from reduced NO bioavailability linked to elevated plasma levels of asymmetric dimethylarginine (ADMA), an endogenous inhibitor of NOS [135,136,137,138]. Despite the abundance of L-arginine as a substrate for NO synthesis, its utilisation can be constrained by NOS uncoupling or interference from ADMA. This phenomenon is a salient example of the so-called 'L-arginine paradox’, underscoring the necessity for targeted interventions to restore NO production [139,140]. The potential benefits of dietary supplementation with L-arginine have been demonstrated through its capacity to alleviate the aforementioned effects, thereby enhancing endothelial function and promoting vasodilation. Nevertheless, it is imperative to exercise caution and meticulously monitor the balance between NO production and the mitigation of adverse effects, such as oxidative stress [10].

**Figure 6:**
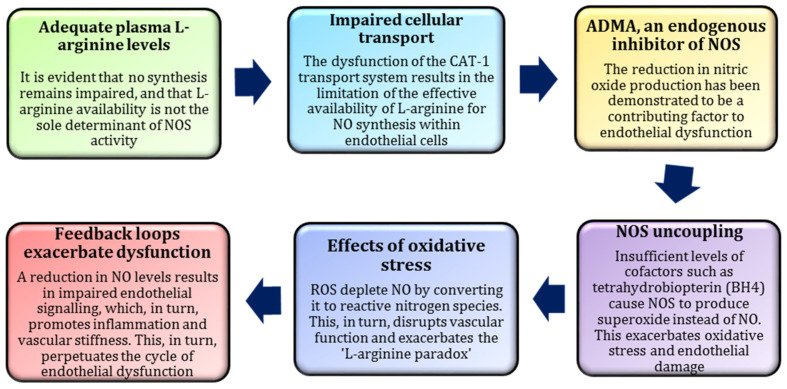
Mechanism of the L-arginine paradox. The L-arginine paradox is defined by the occurrence of impaired nitric oxide production despite adequate or elevated circulating levels of L-arginine. This impairment can be attributed to various factors, including the presence of asymmetric dimethylarginine (ADMA), depletion of such cofactors as tetrahydrobiopterin (BH4), and disturbances in the CAT-1 cellular transport system. This phenomenon highlights the complexity of L-arginine metabolism and underscores the necessity for a multifaceted therapeutic approach.

Research into the effects of L-arginine supplementation in highly trained athletes has yielded inconsistent outcomes. While some studies have indicated no significant enhancement in athletic performance among well-trained individuals, this is probably attributable to the optimisation of NO pathways in such athletes, which already maximises vasodilation and oxygen delivery [141,142,143]. The findings reported by Bescós et al. (2012) suggest that the endogenous NO production in these athletes is sufficient to meet the demands of exercise, rendering additional L-arginine supplementation redundant [144]. A graphical representation of the mechanisms underlying the L-arginine paradox is provided in Figure 6.

The extant research indicates that L-arginine supplementation does not significantly enhance the endurance, strength, or recovery of highly trained athletes, suggesting that their NO production systems already function at optimal capacity [141,142,143,145]. However, additional studies propose that factors such as enhanced mitochondrial efficiency, improved muscle fibre recruitment, and accelerated lactate clearance are more critical contributors to performance improvements in this population [146,147]. Given the extensive cardiovascular and vascular adaptations resulting from long-term training, it is conceivable that these athletes may underutilise supplemental L-arginine [144]. Furthermore, elevated levels of oxidative stress and arginase activity may further limit the bioavailability of L-arginine for NO synthesis, particularly in endurance athletes, where the oxygen delivery demands are persistently high [24,139]. Consequently, the current body of evidence suggests that the benefits of L-arginine supplementation are more pronounced in less-trained individuals or those with such conditions as vascular dysfunction, while its impact remains minimal in highly trained athletes [24,143,148].

### 2.7. Role of Plasma ADMA and Dietary Nitrates

In recent years, there has been increasing interest in the relationship between elevated plasma levels of ADMA and its impact on NO synthesis. ADMA has been demonstrated to competitively inhibit L-arginine binding to NOS, thereby reducing NO production. This inhibition not only diminishes NO synthesis but also increases the generation of superoxide radicals, exacerbating vascular oxidative stress [138,149]. Conversely, nitrate supplementation offers an alternative strategy to restore NO levels by bypassing NOS-dependent pathways. When ingested, dietary nitrates are metabolised into nitrites and subsequently converted to NO under hypoxia, thereby promoting vascular health [150,151]. For individuals who donate blood, the consumption of nitrate-rich foods, such as beetroot and green leafy vegetables, provides a non-invasive method to enhance NO bioavailability while mitigating ADMA-induced NOS inhibition [62,93].

Supplementing blood donors with L-arginine and dietary nitrates may effectively address deficiencies in NO production, thereby improving vascular function and post-donation recovery [152]. L-arginine supplementation has been shown to increase NO bioavailability, counteracting the inhibitory effects of ADMA and enhancing endothelial function [10]. Meanwhile, nitrate supplementation serves as a stable NO reservoir, a property that is particularly advantageous in hypoxic tissues [150,151]. Consequently, these interventions have the potential to reduce endothelial dysfunction and oxidative stress, thereby promoting safer blood donation practices. The implementation of these strategies has the potential to enhance donor health, facilitate recovery, and support long-term donor retention.

It is widely acknowledged that blood donation is a physiological stressor that can induce transient hypoxia or oxygen deprivation [8,153]. This process has the potential to influence the metabolism of nitric oxide and L-arginine [24]. These molecules play pivotal roles in endothelial function and vascular tone regulation [10]. The process of blood donation is associated with a reduction in circulating blood volume and erythrocyte count, which can temporarily diminish oxygen delivery to tissues. In response, the body initiates adaptive mechanisms, including an increased heart rate and vasoconstriction, indicative of physiological stress [154,155]. Furthermore, post-donation reductions in haemoglobin levels have been shown to exacerbate tissue hypoxia, particularly in individuals with low baseline haemoglobin levels or pre-existing health conditions [156,157]. In such contexts, NO synthesis is vital for vascular homeostasis, contributing to the maintenance of vascular tone and the promotion of vasodilation [133]. It is important to note that NO production relies on the availability of its primary precursor, L-arginine [135].

The interplay between stress and hypoxia deserves particular attention. Observations of blood donation have revealed that both conditions are capable of impairing NO production [158,159]. This impairment may stem from reduced L-arginine availability or heightened oxidative stress, which collectively diminish NO bioavailability [158]. A decline in NO production is closely linked to endothelial dysfunction, characterised by impaired vasodilation and reduced vascular responsiveness. This is a critical concern, as endothelial health is essential for maintaining vascular homeostasis and ensuring adequate tissue oxygenation [160].

Recent studies have indicated the occurrence of compensatory mechanisms in response to the physiological stress and hypoxia associated with blood donation. The intricate relationship between endothelial function and NO synthesis remains a focal point of research, particularly concerning the body’s adaptive responses to hypoxic conditions [133]. These adaptive responses frequently entail the upregulation of erythropoiesis and modifications in NO signalling pathways aimed at restoring vascular function [161]. Consequently, it is evident that blood donation induces transient physiological stress and hypoxia, which can disrupt NO and L-arginine metabolism. The reduction in NO production in these conditions may contribute to endothelial dysfunction and impaired vascular regulation [10,133]. A comprehensive elucidation of these mechanisms is crucial for safeguarding donor health and refining blood donation protocols to mitigate potential adverse effects.

### 2.8. Link Between Hypoxia Mechanisms and NO in Blood Donations

Recent research highlights the critical role of NO in regulating vascular tone, blood flow, and oxygen delivery, underscoring its integral involvement in the body’s response to hypoxia [71,159]. In the context of blood donation, NO facilitates blood flow regulation, oxygen distribution, and adaptive responses to hypoxic conditions [162]. A comprehensive examination of the interplay between NO and various hypoxic mechanisms offers significant insights into the recovery processes following blood donation.

The physiological responses to blood donation involve distinct types of hypoxia: acute, moderate, and intermittent [154,155]. Acute hypoxia, which occurs in response to the immediate effects of donation, has been shown to trigger rapid adaptive mechanisms, such as erythropoiesis and metabolic adjustments [163,164]. Moderate hypoxia, prevalent during the recovery phase, promotes longer-term adaptations, including enhanced mitochondrial efficiency and improved oxygen utilisation [165]. Intermittent hypoxia, a concept frequently applied in training paradigms, may also have implications for repeated blood donations, as periodic reductions in oxygen-carrying capacity drive physiological resilience and adaptation to subsequent donations [166,167]. In conclusion, while blood donation does indeed induce transient hypoxia, the body’s adaptive mechanisms—encompassing red blood cell production and optimised oxygen efficiency—enable recovery and the maintenance of homeostasis [153]. These findings emphasise the interplay between hypoxic stress and physiological resilience, offering insights into the dynamic post-donation recovery process.

Research has established a critical link between hypoxic vasodilation and nitric oxide, underscoring the pivotal role of NO in enhancing oxygen delivery during periods of reduced oxygen availability [159,168]. Hypoxic vasodilation, which is mediated by NO, occurs as a result of the release of NO by haemoglobin in response to deoxygenation. This, in turn, leads to vasodilation, which improves blood flow to tissues that are hypoxic. This mechanism ensures efficient oxygen distribution even under constrained supply [169]. Following periods of circulatory stress, such as blood donation, elevated NO production has been observed to augment this adaptive response, facilitating faster recovery [170,171]. Increased NO levels enable the circulatory system to compensate for the reduced oxygen-carrying capacity by improving tissue perfusion and oxygen delivery [170,171].

Furthermore, NO plays a fundamental role in regulating microvascular function, contributing to vascular homeostasis. Such mechanisms as S-nitrosylation (SNO-Hb) in haemoglobin illustrate how NO-dependent pathways ensure adequate oxygen transport [171,172]. Enhanced NO production following blood donation is an adaptive response to circulatory stress, promoting vasodilation and optimising oxygen delivery to hypoxic tissues. This adaptive response not only is a factor in recovery but also highlights the pivotal role of NO in maintaining microvascular equilibrium and facilitating physiological regeneration after blood donation [50,171].

Repeated donations have been shown to induce a state of enhanced NO production and vascular responsiveness within the body, thereby facilitating expedited recovery and optimised adaptation to periodic fluctuations in oxygen delivery [4,81]. Furthermore, NO-mediated enhancements in the efficiency of mitochondrial functioning contribute to the donor’s ability to tolerate hypoxic conditions and accelerate the recovery of red blood cell counts [24,50]. These findings underscore the multifaceted role of NO in adaptive vascular responses and recovery processes in the context of blood donation.

### 2.9. L-Arginine, NO, and Acute Hypoxia

In response to acute hypoxia, the body activates a range of adaptive mechanisms, including the stabilisation of hypoxia-inducible factor-1alpha (HIF-1α) [173]. HIF-1α plays a pivotal role in regulating the cellular response to oxygen deprivation, promoting the production of erythropoietin, a hormone that stimulates erythropoiesis [161]. Under normoxia, the rapid degradation of HIF-1α is catalysed by prolyl hydroxylases (PHDs). However, the decreased oxygen availability during periods of hypoxia results in the inhibition of PHD activity, leading to the accumulation of HIF-1α. Consequently, the accumulation of HIF-1α results in the transcription of target genes associated with erythropoiesis (e.g., erythropoietin) and angiogenesis (e.g., vascular endothelial growth factor, VEGF) [174,175].

**Figure 7:**
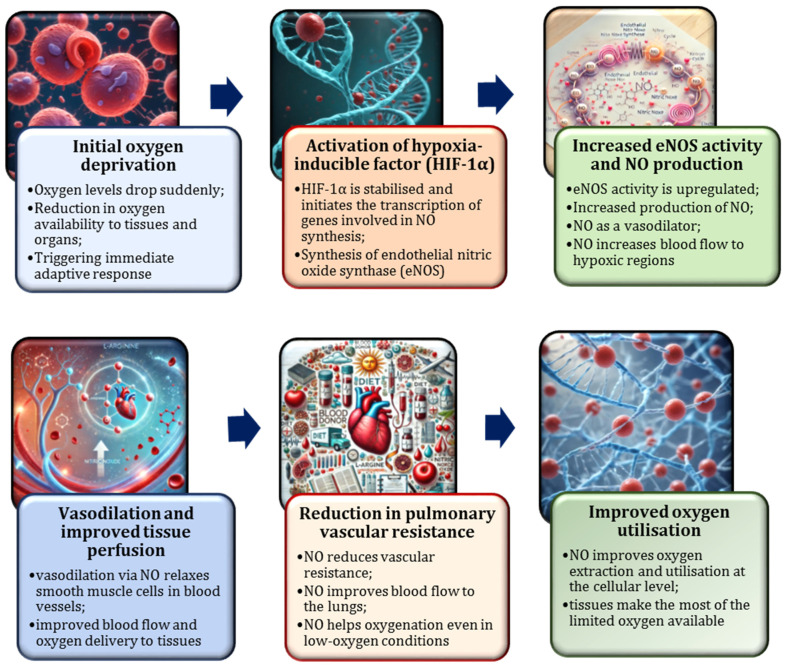
NO and acute hypoxia. Acute hypoxia and NO act as key mediators of vascular function, promoting blood flow, tissue survival, and adaptive responses to changing oxygen levels.

Furthermore, HIF-1α enhances the expression of glycolytic enzymes, such as hexokinase and lactate dehydrogenase (LDH), thereby enabling cells to generate ATP through anaerobic pathways. This metabolic shift supports cellular function in oxygen-limited conditions and facilitates the gradual restoration of blood’s oxygen-carrying capacity, a process that may span several days to weeks [175,176]. Acute hypoxia also induces a temporary shift from aerobic to anaerobic metabolism, mirroring the tissue-level response to the reduced oxygen supply following blood donation [177]. Nitric oxide plays a crucial role in mediating these adaptations [159]. During acute hypoxia, such as that experienced immediately after blood donation when the oxygen-carrying capacity is temporarily diminished due to a reduced red blood cell count, NO production undergoes significant modulation (Figure 7). Understanding the dynamic interplay between acute hypoxia and NO synthesis is fundamental for elucidation of the body’s physiological response to oxygen deprivation.

Numerous studies have explored the critical role of NO in the physiological response to acute hypoxia [159,168,178]. In hypoxic conditions, vascular tissues increase NO production, predominantly mediated by eNOS. This response facilitates the regulation of blood flow and enhances oxygen delivery to tissues. Specifically, the reduction in oxygen availability has been shown to stimulate eNOS activity, resulting in NO release and subsequent vasodilation, thereby optimising perfusion, particularly in tissues experiencing significant oxygen deprivation [3,71]. Following blood donation, the temporary reduction in the red blood cell count induces hypoxia, triggering a compensatory increase in NO production. This mechanism enhances blood flow and ensures the efficient redistribution of the limited oxygen supply, prioritising oxygen delivery to metabolically demanding tissues, such as the brain and muscles [158,179]. Furthermore, NO mitigates ischemic effects by promoting vasodilation and supporting microcirculatory function [4].

Our previous investigations have elucidated adaptive energy supply processes during hypoxia, characterised by rapid succinate accumulation and the simultaneous engagement of dual metabolic pathways: the restoration of Krebs cycle activity and maintenance of α-ketoglutarate oxidation through aminotransferase-mediated reactions [180,181]. Experimental studies involving rats exposed to acute hypoxia (7% oxygen in nitrogen for 30 min) and treated with the NO precursor L-arginine (600 mg/kg) demonstrated variable enhancement in mitochondrial ADP-stimulated respiration. This enhancement was observed when utilising succinate, an FAD-dependent mitochondrial substrate, and α-ketoglutarate, an NAD-dependent substrate for oxidative phosphorylation [180].

These findings suggest that succinate oxidation in the presence of L-arginine represents a short-term adaptive response, improving oxygen utilisation efficiency. Conversely, α-ketoglutarate oxidation was modulated by the NO synthase inhibitor, N^ω^-nitro-L-arginine (L-NNA), which enhanced reductase activity while suppressing NO synthase-dependent pathways. Consequently, nitrite anion oxidation became predominant, accompanied by a decline in urea and polyamine levels. Acute hypoxia was further associated with diminished NO synthase activity during severe tissue oxygen deprivation. This reduction in enzymatic activity was accompanied by increased superoxide anion production and significant activation of lipid peroxidation, underscoring the oxidative stress challenges inherent to acute hypoxia [180,181].

**Figure 8:**
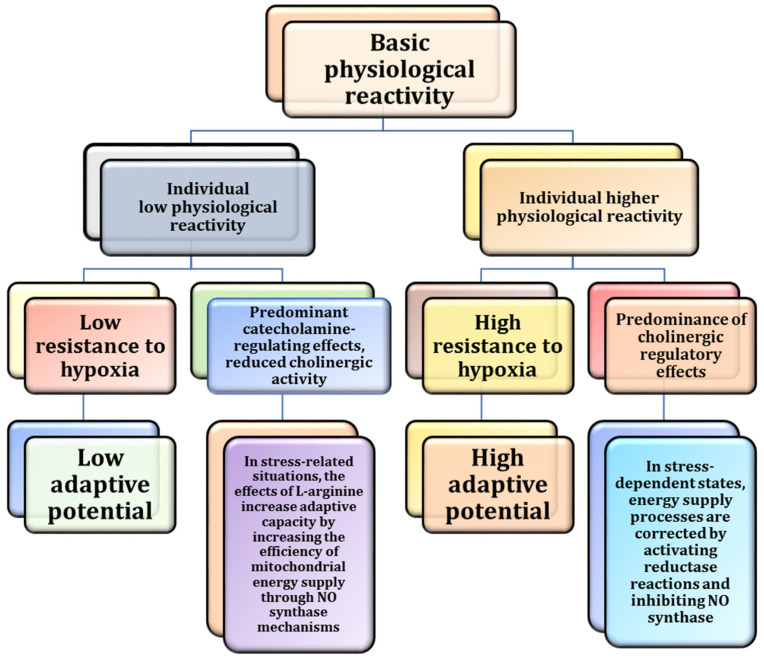
Effects of L-arginine depending on the basic level of the organism’s physiological reactivity and hypoxia resistance factor sensitivity.

It has been demonstrated that the effects of L-arginine as a cholinergic vasoactive factor are dependent on experimental conditions [182]. This is evident when comparing experiments involving guinea pig hearts with those involving rats. This is of particular significance given that guinea pigs exhibit a higher baseline cholinergic status, and nitric oxide exerts some of its effects through acetylcholine receptors. The molecular basis for the dual stimulation of nitric oxide formation as a crucial protective factor in maintaining energy supply during hypoxia is associated with a marked activation of reductase enzymes as opposed to oxygen-dependent oxidase reactions. This shift has been shown to help mitigate the toxic effects of oxygen, superoxide anions, and oxyhemoglobin in hypoxic conditions [24]. It has been shown that targeting NO-generating systems holds great promise for treating various pathological dysfunctions associated with hypoxia [180,181]. These effects have been observed in conditions such as acute hypoxia, stress, dynamic exercise, ionising radiation, and adaptation to interval hypoxic exercise [181]. For example, L-arginine preserved NAD-dependent mitochondrial oxidation under stress but depleted respiratory reserves, reflecting stress-induced shifts in mitochondrial activity driven by elevated catecholamine levels [181]. This mechanism is particularly relevant in models where L-arginine is administered, either in animal studies or as a supplement in humans. The effects are pronounced in individuals or species with high cholinergic regulatory mechanisms, reflecting greater adaptive reserves. For example, guinea pigs (compared to Wistar rats) [182] or highly trained athletes (compared to beginners) show increased resistance to hypoxic factors [183,184]. In such cases, improved mitochondrial function and reduced production of ROS correlate with a reduced role of NO synthase mechanisms, a reciprocal increase in nitrite reductase activity, and an effective reduction in ROS formation [181]. These relationships are illustrated in Figure 8.

### 2.10. Moderate Hypoxia and NO

A frequently under-explored facet of the nitric oxide research pertains to its function during moderate hypoxia, a condition that arises during the recovery phase subsequent to blood donation and encompasses protracted periods of low oxygen levels. In this state, the body activates long-term adaptive mechanisms, such as enhanced mitochondrial function, improved oxygen utilisation, and increased red blood cell production through erythropoiesis [185]. In contrast to the acute form of hypoxia, which elicits a swift and pronounced response, moderate hypoxia is distinguished by the sustained activation of HIF-1α at lower oxygen levels. This sustained activation facilitates a series of processes that enable the body to adapt to reduced oxygen levels. These include an increase in mitochondrial density, enhanced angiogenesis, and optimisation of energy production pathways to maintain cellular efficiency despite oxygen deprivation [186,187].

A significant adaptation during moderate hypoxia is mitochondrial biogenesis, where the number of mitochondria increases, thereby enhancing the cell’s ability to generate ATP, even in low-oxygen conditions. This process is subject to regulation by the PGC-1α (peroxisome proliferator-activated receptor gamma coactivator 1-alpha) pathway, which controls genes involved in mitochondrial function and biogenesis [188,189]. While moderate hypoxia is less severe than acute hypoxia, it can persist, particularly in blood donors with low iron levels or slower erythrocyte regeneration [118,163].

**Figure 9:**
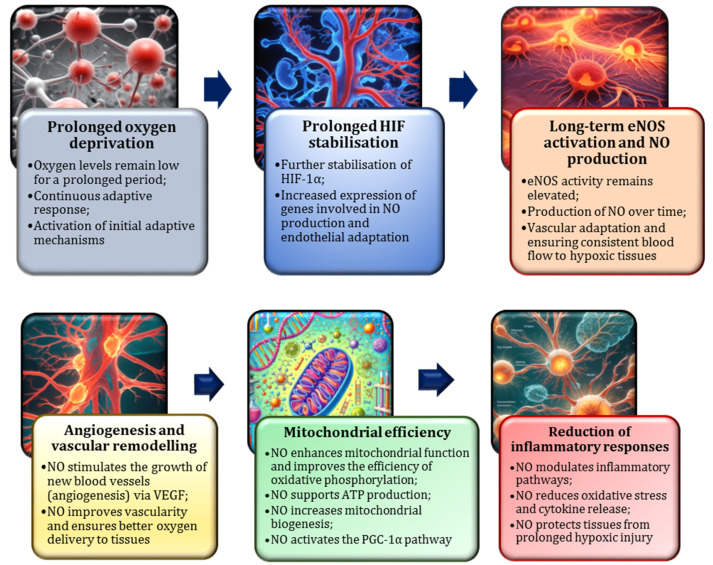
The effect of nitric oxide in moderate hypoxia. NO production results in a more gradual adaptation to oxygen deprivation. VEGF—vascular endothelial growth factor, PGC-1α—peroxisome proliferator-activated receptor gamma coactivator 1α.

During this phase, NO continues to play a critical role in supporting blood flow and facilitating adaptive responses [4]. The mechanism of action of nitric oxide in moderate hypoxia differs from that in acute hypoxia. While NO remains essential for vasodilation and oxygen delivery, it also influences mitochondrial function by enhancing respiration in low-oxygen conditions [71,171]. Research indicates that NO interacts with mitochondrial enzymes, such as cytochrome c oxidase, to improve mitochondrial efficiency and ATP production during hypoxic stress [190,191]. Furthermore, NO has been demonstrated to promote the expression of angiogenesis-related genes, such as VEGF, thereby stimulating the formation of new blood vessels and enhancing long-term oxygen delivery [192]. The role of NO in moderate hypoxia is depicted in Figure 9.

In the aftermath of blood donation, a state of moderate hypoxia ensues as the body undergoes a process of recovery. Nitric oxide has been demonstrated to play a pivotal role in compensating for the reduced red blood cell count by sustaining vasodilation, which enhances blood flow and improves tissue oxygenation [171]. Furthermore, NO has been demonstrated to promote angiogenesis and mitochondrial adaptations, thereby assisting the body in more efficaciously managing lower oxygen levels during the recovery process [193]. This NO-driven response is crucial for facilitating healing and restoring normal oxygen-carrying capacity, particularly in tissues most impacted by the donation [193].

Moderate hypoxia instigates adaptive processes that increase the cellular oxygen utilisation efficiency. These adaptations include the upregulation of enzymes within the electron transport chain, such as cytochrome c oxidase, which supports enhanced oxygen consumption and ATP production in low-oxygen conditions [173,194]. A key aspect of this adaptation is the stabilisation of redox homeostasis. While moderate hypoxia can result in elevated ROS production, the body counteracts this condition by activating antioxidant pathways. This balance is critical for protecting cells from oxidative damage and ensuring their proper function, even under hypoxic stress [195].

### 2.11. L-Arginine, NO, and Intermittent Hypoxia

Recent studies have emphasised the significance of intermittent hypoxia training (IHT), which involves periodic exposure to low oxygen levels [166,167,181], as a valuable model for understanding how NO facilitates adaptation to repeated cycles of blood loss and recovery, such as those encountered by frequent blood donors. During IHT, each hypoxic episode induces an increase in NO production, promoting vasodilation and enhancing oxygen delivery. Repeated hypoxic exposures over time result in a more efficient NO response, characterised by elevated NOS activity and improved vascular function [159,167,181,196].

It has been observed that IHT triggers adaptive processes analogous to those observed in moderate hypoxia, including the activation of HIF-1α and PGC-1α [197,198]. These mechanisms enhance mitochondrial efficiency, stimulate red blood cell production, and optimise overall oxygen utilisation. Furthermore, NO contributes to maintaining mitochondrial efficiency during IHT, thereby supporting aerobic metabolism even in low-oxygen conditions [181]. The repeated activation of HIF-1α during IHT has been shown to promote genetic adaptations that enable the body to better tolerate fluctuating oxygen levels [173]. Furthermore, chronic HIF-1α activation has been demonstrated to enhance performance in hypoxic conditions by stimulating erythropoietin production, mitochondrial biogenesis, and angiogenesis [163,173].

Repeated cycles of hypoxia have been demonstrated to stimulate mitochondria, thereby enhancing their capacity for energy production. This process is characterised by augmentation in mitochondrial density and enhancement in the efficiency of oxidative phosphorylation, consequently augmenting the cellular energy output and optimising oxygen utilisation [199]. In the context of frequent blood donation, this process is analogous to intermittent hypoxia, where the body is periodically exposed to low oxygen levels following each donation, followed by recovery periods [8,154]. The trajectory of haemoglobin mass and erythropoietin levels indicates that the regenerative capacity of total haemoglobin mass declines with repeated blood donations. Specifically, the reduction in haemoglobin mass is preceded by decreases in haematocrit, haemoglobin concentration, erythrocyte count, and ferritin levels [200].

**Figure 10:**
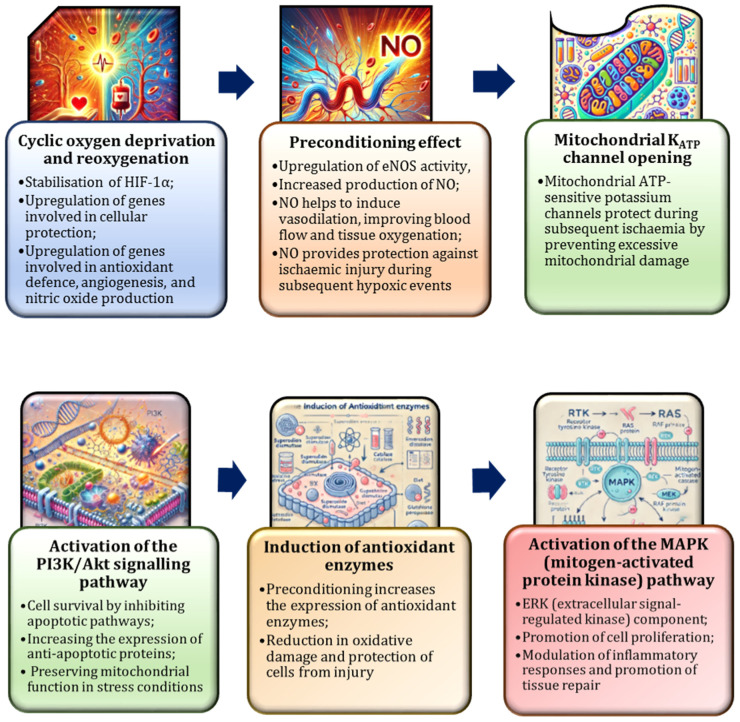
Intermittent hypoxia and NO. Molecular mechanisms involved in the preconditioning effect that make tissues more resistant to future damage following brief or intermittent exposure to stressors such as hypoxia, and these mechanisms together contribute to the protective effects of preconditioning by increasing tissue tolerance to subsequent periods of ischaemia or hypoxia. PI3K/Akt Pathway—phosphoinositide 3-kinase/protein kinase B.

Our study has highlighted the significant anti-stress effects of L-arginine and IHT, both of which effectively enhance the body’s compensatory mechanisms in stressful conditions [181]. By modulating the NO system, L-arginine facilitates mitochondrial adaptation and reduces lipoperoxidation, offering critical protection against oxidative cellular damage. Furthermore, IHT activates reserves within the catecholaminergic and cholinergic systems, thereby amplifying the body’s adaptive responses to hypoxia and stress [181]. Our study examined the effects of L-arginine, IHT, and acute emotional stress models on oxygen-dependent processes in rats, with a focus on mitochondrial oxidative phosphorylation, microsomal oxidation, and the intensity of lipoperoxidation. The role of NOS in the regulation of catecholamines, including epinephrine, norepinephrine and their precursors DOPA and dopamine, and cholinergic activity, measured by acetylcholine and acetylcholinesterase activity, was also investigated [181]. As shown in Figure 10, L-arginine plays a key role in the production of NO. The figure illustrates how intermittent hypoxia, a condition characterised by fluctuations in oxygen levels, can reduce NO bioavailability by increasing oxidative stress. However, supplementation with L-arginine has been shown to help preserve endothelial function and promote vascular relaxation. This in turn has the potential to attenuate the cardiovascular consequences of intermittent hypoxia.

### 2.12. NO and Blood Donation

The hypothesis under consideration is that the production of NO increases with each blood donation, thereby enhancing blood flow and oxygen delivery during the recovery phase. The findings reported by Premont et al. (2020) provide indirect support for this hypothesis, suggesting that elevated NO production improves vascular function during recovery [171]. NO plays a pivotal role in hypoxic vasodilation, as it is released from haemoglobin in proportion to the degree of deoxygenation, thus promoting efficient vasodilation to meet tissue oxygen demands [169,171]. Increased NO production during blood donation may amplify this mechanism, particularly in hypoxic conditions, improving the efficiency of oxygen transport and facilitating faster recovery. Furthermore, the study conducted by Premont et al. (2020) emphasises the critical role of NO in maintaining microvascular health [171]. Through S-nitrosylation dynamics (SNO-Hb), the release of NO from haemoglobin regulates microcirculatory flow, a process that is essential for oxygen delivery and tissue recovery [201]. The enhanced NO production during blood donation may represent an adaptive response, promoting vasodilation and aiding in the restoration of homeostasis following transient circulatory stress. This underscores the pivotal role of NO in ensuring optimal oxygen delivery and post-donation recovery efficiency.

The relationship between endothelial function and NO synthesis remains a significant area of research, particularly with regard to the role of L-arginine in mitigating lipid peroxidation in various stress-related conditions and pathologies [10,24]. As a precursor of NO, L-arginine plays a crucial role in reducing lipid peroxidation, which serves as a primary indicator of oxidative damage [24,59,181]. The lipid peroxidation process is observed when ROS attacks polyunsaturated fatty acids in cell membranes, resulting in the generation of malondialdehyde (MDA) and other harmful by-products [202]. L-arginine, a precursor of NO synthesis via eNOS, functions as an antioxidant by neutralising ROS and thereby interrupting the chain reactions that drive lipid peroxidation [24,59,181]. Consequently, enhanced bioavailability of NO contributes to the reduction in oxidative stress, thus safeguarding cell membranes and preserving their structural and functional integrity [76,86].

A further critical aspect of NO research is to understand how L-arginine mitigates microsomal oxidation in stress-related conditions, as highlighted in recent studies [59,181,203]. Microsomal oxidation, frequently facilitated by cytochrome P_450_ enzymes, generates ROS, which contribute to oxidative damage. Evidence suggests that this system accounts for up to 40% of oxidative processes [204,205]. L-arginine counteracts this damage through two primary mechanisms: enhancement in NO production and modulation of antioxidant system activity. The NO generated by L-arginine has been shown to inhibit NADPH oxidase, a significant source of ROS in microsomal systems [50,206]. Furthermore, L-arginine has been shown to support cellular antioxidant defences by promoting glutathione synthesis, a crucial intracellular antioxidant, and activating the Nrf2 pathway [207]. Collectively, these mechanisms reduce ROS production, preserve microsomal membrane integrity, and prevent the leakage of oxidised lipids and proteins into the cytoplasm [59,181,203].

### 2.13. L-Arginine and Donor Diet

This study highlights the potential synergistic effects of L-arginine supplementation combined with an antioxidant-rich diet, particularly in the context of blood donation. The integration of L-arginine with dietary antioxidants has been demonstrated to enhance the protective response to oxidative stress, thereby supporting the well-being of blood donors [206,208]. Specifically, dietary antioxidants, i.e., vitamins C and E, polyphenols, and others directly neutralise ROS, complementing the ROS-reducing actions of NO derived from L-arginine. Specifically, as demonstrated by Mortensen et al. (2014), vitamin C aids in the regeneration of oxidised NO, thereby increasing its bioavailability [209]. In addition, vitamin E prevents lipid peroxidation in cell membranes, as evidenced by Niki (2021) [210]. Polyphenols, with their established anti-inflammatory and antioxidant properties, support endothelial function and enhance NO synthesis [211,212,213]. Collectively, these compounds work synergistically with L-arginine to offer robust protection against oxidative damage and improve cellular resilience.

Recent studies have also explored the role of dietary nitrate as an alternative pathway for NO production [151]. Dietary nitrate, found in vegetables such as beetroot and spinach, serves as an important source of NO, particularly in conditions of low oxygen levels or high acidity, as this can impair eNOS activity [62]. This alternative pathway has been shown to be of particular benefit to the elderly and individuals with impaired NO synthesis due to various pathological conditions [214]. The conversion of nitrates to nitrites and subsequently to NO is facilitated by oral and intestinal microbiota. Consequently, the combination of L-arginine and dietary nitrate ensures consistent NO availability, even when one pathway is less effective [214]. This dual pathway approach is especially advantageous during oxidative stress, as NO from both sources promotes vascular health, reduces lipid peroxidation, and enhances the antioxidant defence system, thereby preserving cellular integrity [50,215].

It is important to note that blood donation can induce a reduction in blood volume and haemoglobin levels, leading to a temporary decrease in the oxygen-carrying capacity of blood and subsequent tissue hypoxia [154]. Nitric oxide has been identified as playing a central role in the physiological response to this condition by regulating blood flow, oxygen delivery, and vascular tone [171]. Subsequent to donation, the body relies on L-arginine metabolism to produce NO, facilitating adaptation to reduced oxygen availability [95].

The haemodynamic and metabolic alterations occurring after blood donation are crucial for the recovery process [216,217]. The initial reduction in oxygen delivery activates compensatory mechanisms aimed at restoring homeostasis. A significant response to this decrease is NO-mediated vasodilation, which enhances blood flow and compensates for the reduced oxygen-carrying capacity. The availability of L-arginine, which is metabolised by NOS to produce NO, plays a critical role in this process. Vasodilation is critical for ensuring adequate oxygen delivery to vital organs, such as the brain and muscles, during the immediate post-donation period [27,171].

Long-term blood donors undergo transient changes in oxygen delivery and vascular adaptations throughout the recovery phase. To restore normal oxygen levels, the body increases erythropoietin production, stimulating red blood cell synthesis to replace those lost [154,218]. Concurrently, the vascular system undergoes adaptation by enhancing NO production to ensure optimal blood flow. This process involves the continued metabolism of L-arginine to NO via endothelial nitric oxide synthase. The resulting increase in NO availability supports vasodilation and facilitates the redistribution of blood flow to critical tissues, ensuring effective oxygen delivery as red blood cell levels are replenished [3,47].

### 2.14. Role of L-Arginine and NO in Post-Donation Recovery

The mobilisation of L-arginine reserves and the upregulation of eNOS activity are critical elements of donor physiology. The body’s ability to maintain NO synthesis in the conditions of elevated physiological demand is contingent on the effective mobilisation of intracellular L-arginine stores and the activation of eNOS [4]. L-arginine is stored in intracellular compartments, such as the cytosol and lysosomes, with its availability regulated by specific transport systems, notably the cationic amino acid transporter-1 (CAT-1) [219]. During vascular stress or increased activity, eNOS activity is increased, typically in response to such stimuli as shear stress and the activation of signalling pathways, including PI3K/Akt [220]. These adaptive mechanisms ensure that the NO production aligns with physiological needs for vasodilation and tissue oxygenation.

Despite the well-established role of NO in vascular function, ongoing research continues to investigate strategies for optimising its production through dietary and pharmacological interventions, particularly regarding its contribution to vascular elasticity and haemodynamic stability [4]. It has been established that NO plays a pivotal role in modulating the relaxation of vascular smooth muscle cells, thus promoting the dynamic adaptability of blood vessels to fluctuations in blood flow and pressure [4]. By binding to soluble guanylyl cyclase (sGC) in smooth muscle cells, NO increases cyclic guanosine monophosphate (cGMP) levels, inducing relaxation and enhancing vascular compliance. This enhanced elasticity not only alleviates cardiovascular stress but also facilitates efficient blood flow during periods of physical activity or stress [50,71]. Furthermore, NO contributes to haemodynamic stability by ensuring even blood distribution across the vascular network, preventing localised hypoxia and supporting systemic oxygen delivery [159,171].

It is imperative to comprehend the ramifications of L-arginine deficiency or impaired NO synthesis, particularly within the context of donor fatigue and recovery. A lack of L-arginine or impaired NO production can result in significant physiological disruptions, such as diminished vasodilation, compromised oxygen and nutrient delivery, and inefficient removal of metabolic by-products. These deficits can manifest as increased fatigue, muscle soreness, and prolonged recovery periods [1]. Furthermore, insufficient NO levels have been demonstrated to exacerbate oxidative stress, thereby hindering recovery processes and overall physiological resilience [221]. These findings underscore the pivotal role of L-arginine in NO synthesis and its impact on vascular health and recovery, particularly among individuals who engage in regular physical activity or frequent blood donation. Consequently, ensuring sufficient L-arginine availability is imperative to optimise recovery and maintain performance [1,222].

Another critical issue is the risk of hypoperfusion or suboptimal vascular responses following exercise or blood donation [223]. Impaired NO synthesis increases the likelihood of hypoperfusion, a condition marked by insufficient blood flow to tissues during or after exercise. This can lead to localised ischaemia, cellular damage, and delayed recovery [185]. The absence of NO-mediated vasodilation reduces the ability of blood vessels to dynamically meet the elevated demand for oxygen and nutrients, particularly in skeletal muscle. Over time, chronic deficiencies in NO production may result in maladaptive cardiovascular outcomes, including reduced vascular responsiveness and compromised endothelial function [81]. Future research should aim to further explore these risks, underscoring the importance of L-arginine and NO in maintaining vascular health and supporting effective physiological adaptation during periods of stress or recovery.

### 2.15. Dietary Strategies for Blood Donors

Whilst the extant evidence provides a robust foundation, it is imperative to consider additional factors, particularly the nutritional needs for the rapid recovery of blood donors. It is acknowledged that blood donation imposes considerable physiological demands on the donor, thus necessitating the implementation of bespoke nutritional strategies to facilitate recovery and maintain optimal health. Nutritional interventions are crucial in replenishing essential nutrients, reducing oxidative stress, and promoting vascular and tissue repair. A balanced diet, abundant in vital vitamins, minerals, and amino acids, has been demonstrated to enhance recovery outcomes and assist in maintaining donor health between donations [17].

The integration of foods abundant in L-arginine, such as poultry, fish, soy, and pumpkin seeds, is imperative for the promotion of nitric oxide synthesis [36]. These foods have been demonstrated to facilitate endothelial repair and contribute to the maintenance of cardiovascular health. The significance of L-arginine-rich foods in NO synthesis cannot be overstated. The key sources of L-arginine include turkey breast (~2.5 g/100 g), chicken breast (~2.1 g/100 g), pumpkin seeds (~2.3 g/100 g), soybeans (~2.2 g/100 g), peanuts (~3.0 g/100 g), and spirulina (~2.7 g/100 g). Other notable sources are cooked tuna (~1.7 g/100 g) and salmon (~1.2 g/100 g). In contrast, foods such as fruits (e.g., apples and bananas, ~0.02–0.05 g/100 g), vegetables (e.g., cucumbers, ~0.04–0.1 g/100 g), and refined cereals (e.g., white rice, ~0.1–0.15 g/100 g) contain minimal amounts of L-arginine. Data from the USDA Food Composition Database [224] and peer-reviewed studies [225,226,227,228] emphasise the importance of prioritising protein-rich foods over lower-arginine options, such as fruits and refined cereals, to optimise vascular health and recovery. Consequently, the integration of L-arginine-rich foods into a nutritionally balanced diet has the potential to substantially augment recovery from the physiological stress associated with blood donation. This approach offers broader benefits, including enhanced immune function and reduced oxidative damage [17,26].

**Figure 11:**
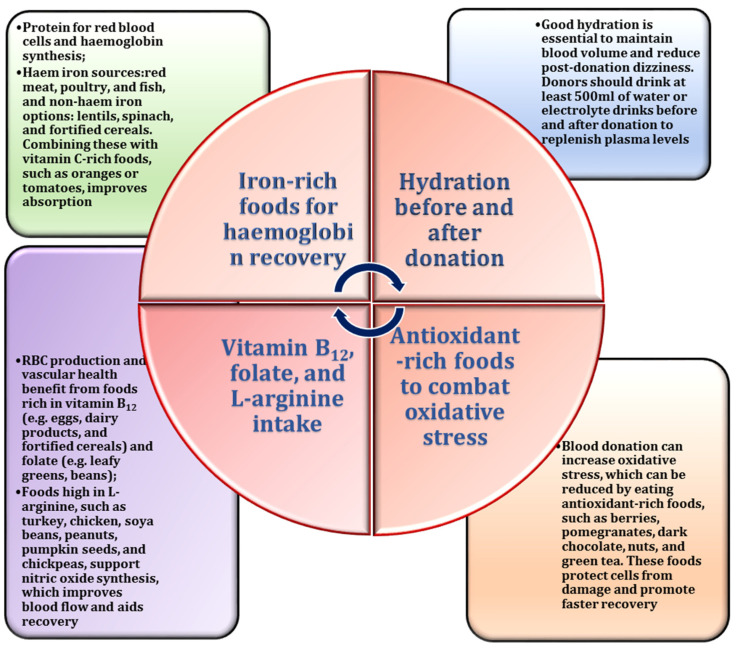
Nutritional strategies for blood donors. Incorporating these strategies, particularly foods rich in L-arginine, can help optimise recovery and reduce the risk of rejection. RBC—red blood cell.

It is imperative to emphasise nutrients that mitigate red blood cell depletion, promote NO production, and accelerate tissue regeneration [26]. A comprehensive understanding of these dietary components facilitates more effective guidance tailored to the specific needs of blood donors. Current research indicates that rapid recovery following donation is contingent upon the replenishment of lost nutrients and the support of physiological systems [229]. Specifically, iron, a critical component of haemoglobin, is among the most essential nutrients for post-donation recovery [230]. Insufficient iron intake has been demonstrated to impair the body’s capacity to produce new red blood cells and restore the oxygen-carrying capacity [18]. Furthermore, protein-rich foods containing essential amino acids are vital for tissue repair and immune function [231]. Adequate hydration is another key aspect of recovery, as it helps maintain blood volume and alleviates post-donation symptoms, such as dizziness and fatigue [232]. It is imperative to note that certain substances should be avoided post-donation. Specifically, caffeine and alcohol should be limited due to their diuretic effects, which may exacerbate dehydration and delay plasma replenishment. Instead, donors should be encouraged to focus on rehydration with water, herbal teas, or electrolyte-rich drinks [17]. The incorporation of L-arginine-rich foods is a particularly salient strategy that can optimise recovery and support vascular and cellular function following donation (Figure 11).

The essential role of iron-rich foods and vitamin C in post-donation recovery has been thoroughly examined by researchers [230,233,234]. Iron-rich foods, including red meat, spinach, and lentils, are found to be instrumental in replenishing haemoglobin levels following blood donation [230]. These foods provide both haem and non-haem iron, which are vital for red blood cell production [18]. To enhance iron absorption, it is particularly beneficial to pair these foods with vitamin C-rich sources, such as citrus fruits, peppers, and tomatoes [235]. Vitamin C facilitates the reduction of ferric iron to ferrous iron, thereby increasing its bioavailability in the gastrointestinal tract [18]. This synergistic dietary approach is of particular importance for donors at risk of iron deficiency, as it facilitates the efficient restoration of haemoglobin levels and energy reserves [17].

### 2.16. Key Nutrients to Support the NO Pathway

Recent studies have highlighted the crucial function of antioxidants in maintaining the stability and bioavailability of NO by mitigating ROS that degrade NO. Vitamin C has been shown to be particularly effective in neutralising superoxide radicals, which, in the absence of this intervention, would react with NO to form peroxynitrite—a detrimental compound that disrupts endothelial function [100]. Research carried out by Taddei et al. (1998) [236] and Mortensen and Lykkesfeldt (2014) [209] demonstrated that vitamin C supplementation enhances NO bioavailability, thereby promoting vasodilation and improving endothelial health. In a similar manner, vitamin E has been shown to protect lipid membranes from oxidative damage, thereby reducing conditions that foster ROS production [210]. Collectively, these antioxidant functions are synergistic in preserving NO levels, ensuring optimal vascular and metabolic function, particularly during periods of oxidative stress, such as those induced by exercise or blood donation [210]. The collective antioxidant effect underscores the significance of incorporating antioxidant-rich foods into dietary strategies to support NO function and overall donor well-being [17,237].

In addition to antioxidants, dietary nitrates—found in such foods as beetroot and leafy greens—offer an alternative pathway for NO synthesis that is independent of L-arginine. These nitrates are converted into nitrites by oral bacteria, which are subsequently transformed into NO in hypoxic conditions, thus bypassing the need for enzymatic NOS activity [62,238]. As suggested by Olas (2024), diets rich in nitrates enhance vascular elasticity and lower blood pressure by increasing NO production through this alternative pathway [64]. In particular, beetroot juice has gained recognition for its effectiveness in improving exercise performance and recovery, making it a promising dietary intervention for blood donors [239]. This nitrate-dependent mechanism is particularly advantageous for individuals with impaired NOS function or limited L-arginine availability [238].

In addition, emerging evidence suggests that magnesium and potassium—electrolytes that play a key role in the body—have a significant impact on vascular health and the NO pathway. Specifically, magnesium modulates NOS activity and decreases vascular resistance, thereby supporting endothelial function [240]. Research conducted by Kostov and Halacheva (2018) indicates that magnesium deficiency impairs NO-mediated vasodilation, heightening the risk of hypertension and vascular stiffness [241]. Conversely, potassium has been shown to regulate blood pressure by balancing sodium levels and maintaining the electrochemical gradient necessary for smooth muscle relaxation [242]. Collectively, these minerals enhance NO efficacy in promoting vasodilation and maintaining haemodynamic stability, underscoring their importance for individuals recovering from blood donation [243]. The collective effect of antioxidants, nitrates, and electrolytes is synergistic, optimising NO production and function [26].

The recent decades have seen a growing emphasis in the research on the potential of antioxidant-rich spices, herbal supplements, and adaptogens to mitigate oxidative stress resulting from excessive NO production or hormonal imbalances [244,245]. These dietary and lifestyle practices provide a holistic approach to enhancing hormonal balance, optimising NO production, and supporting vascular health in blood donors [17]. Spices such as turmeric, ginger, and cinnamon contain powerful antioxidants that neutralise free radicals, thereby reducing oxidative damage to blood vessels and improving vascular function [246,247,248]. Specifically, the active compound in turmeric, i.e., curcumin, has been shown to enhance eNOS activity, thereby promoting NO synthesis and reducing inflammation [249]. Ginger is well-recognised for its anti-inflammatory and vasodilatory effects, which support blood flow and alleviate oxidative stress [250]. Furthermore, cinnamon has been found to improve insulin sensitivity and lower cortisol levels, offering benefits for individuals managing insulin resistance and stress [251].

Adaptogenic herbs, including ashwagandha, rhodiola, and holy basil, have been shown to offer additional benefits by modulating cortisol levels and promoting overall hormonal balance [252]. Ashwagandha, in particular, has been shown to elevate testosterone levels while reducing cortisol, thereby mitigating stress-induced vascular dysfunction [253,254]. Holy basil plays a role in regulating insulin, making it especially beneficial for individuals at risk of metabolic disorders or insulin resistance [255,256]. These herbs enhance NO synthesis by improving vascular function and address hormonal imbalances that can hinder NO production [254,256]. Consequently, the integration of these dietary and lifestyle interventions into recovery protocols for blood donors can facilitate comprehensive support for vascular health and overall well-being [17].

### 2.17. Nitric Oxide, Blood Donation, Potential Risks, and Limitations

Excessive NO production, influenced by dietary factors, may pose a risk to blood donors by inducing hypotension through excessive vasodilation, as evidenced by Gamboa et al. (2008) [257]. The ingestion of nitrate-rich sources, such as beetroot and leafy vegetables, and amino acids like L-arginine found in poultry, fish, and soy, has been demonstrated to elevate NO levels, potentially resulting in enhanced vasodilation [36,62]. While vasodilation generally improves blood flow and vascular function, excessive NO levels can result in a significant drop in blood pressure, particularly in donors already compromised by blood loss [257]. Consequently, the dietary intake of NO-promoting foods necessitates meticulous post-donation monitoring to avert deleterious outcomes, particularly in donors with a propensity for hypotension or a history of cardiovascular instability [17].

Imbalanced NO production can also lead to the formation of reactive nitrogen species (RNS), such as peroxynitrite, which causes oxidative damage to tissues [85]. Antioxidant-rich foods, including those containing vitamins C and E (e.g., citrus fruits, nuts, and seeds), play a crucial role in mitigating oxidative stress [258]. However, excessive NO production induced by diets high in nitrates or L-arginine may overwhelm the body’s antioxidant defences and exacerbate oxidative damage caused by RNS [259]. This oxidative stress can impair endothelial function, hinder tissue repair, and delay recovery in blood donors [10,15,17,18,25]. Consequently, a diet deficient in antioxidants may increase the risk of endothelial dysfunction and prolong recovery following blood donation.

As the understanding of vascular health advances, there is growing interest in how nitrate-rich diets, such as those containing beetroot, may potentiate the effects of medications like nitrates or phosphodiesterase inhibitors, potentially leading to hypotension [62,260]. Furthermore, individuals with such conditions as hypertension or diabetes, which can alter NO bioavailability, may exhibit different vascular responses to dietary changes [261,262]. In such cases, a meticulously balanced diet comprising L-arginine-rich foods (e.g., fish and nuts) and antioxidant-rich foods may assist in NO production and maintain vascular health. However, it is imperative to emphasise that dietary recommendations must be tailored to the individual’s health status, taking into account their medication regimen and underlying medical conditions, to avoid potential adverse interactions [263].

### 2.18. Hormones and Nitric Oxide Interactions

The role of oestrogen in regulating NO production in women is well-documented, with the enhancement of eNOS activity being a key mechanism. This results in increased NO synthesis in blood vessels. This effect is mediated through the interaction of oestrogen with oestrogen receptors (ERs) located on endothelial cells, thereby activating downstream signalling pathways, including the PI3K/Akt pathway [264]. The activation of these pathways has been shown to upregulate eNOS expression, thereby improving vascular function and promoting vasodilation [265]. Miller and Duckles (2008) have demonstrated that the contribution of oestrogen to enhanced NO production is essential for optimal blood flow and vascular health, particularly during the reproductive years [266]. However, post-menopausal women experience a decline in oestrogen levels, leading to reduced NO production and an increased risk of endothelial dysfunction and cardiovascular diseases [267].

Testosterone, the primary male sex hormone, also plays an important role in NO production, though via different mechanisms from those of oestrogen. Testosterone has been shown to interact with androgen receptors on endothelial cells, thereby stimulating the phosphorylation of eNOS and, consequently, NO synthesis [268,269]. Shoskes et al. (2016) demonstrated that testosterone supplementation improved endothelial function in hypogonadal men, suggesting its positive impact on NO bioavailability [270]. However, it is important to note that excessive testosterone levels can lead to adverse cardiovascular outcomes, emphasising the importance of hormonal balance for maintaining optimal NO production [271,272].

Insulin exerts a multifaceted influence on NO production, exhibiting both beneficial and detrimental effects, which are contingent upon its concentration and the metabolic state of the body. Insulin has been shown to enhance eNOS activity by activating the PI3K/Akt pathway, thereby promoting NO production in endothelial cells [273]. This mechanism is critical in glucose regulation and vascular health. However, in individuals with insulin resistance or diabetes, the efficacy of this pathway is impaired, resulting in reduced NO production and endothelial dysfunction [274]. Chronic hyperinsulinaemia and insulin resistance, often associated with obesity and metabolic syndrome, further reduce NO bioavailability, exacerbating impaired vasodilation, hypertension, and cardiovascular risk [275].

Research by Manrique et al. [276] showed that vascular insulin sensitivity was impaired, which is an early defect leading to impaired vascular relaxation. This defect is common in overweight, obese, and hypertensive individuals, where it is associated with systemic and vascular insulin resistance. Resistance, as well as activation of the renin–angiotensin–aldosterone system, with activated angiotensin II type 1 receptor and mineralocorticoid receptor signalling, further promotes the development of vascular insulin resistance and impaired endothelial NO-mediated relaxation [276].

The effect of cortisol, a stress hormone, on NO production is also noteworthy. Elevated cortisol levels, particularly during chronic stress, have been shown to inhibit eNOS activity by suppressing endothelial function and inducing oxidative stress. This interaction is mediated by glucocorticoid receptors (GRs) in endothelial cells, resulting in reduced NO synthesis and increased vascular stiffness [86,277,278]. Furthermore, chronic elevations in cortisol, as observed in conditions such as Cushing’s syndrome or prolonged stress, have been demonstrated to impair vasodilation, increase blood pressure, and contribute to endothelial dysfunction [279]. In a study conducted by Sher et al. (2020) [280], it was demonstrated that cortisol-induced suppression of eNOS significantly reduces NO availability, thereby impairing vascular adaptability. Consequently, effective management of cortisol levels through stress-reduction strategies and the maintenance of a balanced lifestyle are imperative for preserving NO production and vascular health [280].

Thyroid hormones, including thyroxine (T4) and triiodothyronine (T3), have also been demonstrated to regulate NO production by modulating endothelial function and vascular tone [281]. These hormones have been shown to enhance eNOS expression and activity, thereby promoting NO-mediated vasodilation [281]. As highlighted by Gluvic et al. (2020) [282], thyroid hormone insufficiency, such as hypothyroidism, reduces NO availability and impairs vascular reactivity. Conversely, hyperthyroidism has been observed to result in excessive NO production, leading to vasodilation and vascular endothelial damage, and is identified as a significant risk factor for cardio-cerebrovascular diseases [283]. The delicate balance of thyroid hormones underscores their central role in regulating NO production and supporting vascular health [281,284].

### 2.19. Lifestyle and Dietary Strategies in Blood Donors

This section emphasises the pivotal function of dietary modifications in promoting hormonal equilibrium and overall health, particularly among blood donors. A nutrient-dense diet has been shown to significantly enhance proper hormonal regulation, particularly in relation to NO production. Specifically, the consumption of healthy fats, such as those found in olive oil, avocados, and oily fish (which are rich in omega-3 fatty acids), has been demonstrated to regulate oestrogen and testosterone levels, given the influence of lipid metabolism on these hormones [285,286]. Magnesium-rich foods, including leafy greens, nuts, and seeds, play a dual role in supporting thyroid function and managing cortisol levels while also contributing to vascular health by supporting NO production [287]. Furthermore, whole grains and lean proteins have been shown to enhance metabolic health, a crucial factor in maintaining insulin sensitivity and preventing the onset of insulin resistance, a condition that has been demonstrated to impede NO synthesis [288]. It is particularly beneficial for blood donors to make lifestyle and dietary changes that address hormonal imbalances influencing NO production and vascular health. The integration of nutrient-rich foods, regular physical activity, and antioxidant-rich spices in the diet of donors has been shown to accelerate recovery, enhance vascular function, and optimise post-donation NO bioavailability [17].

Regular physical activity is increasingly being recognised as a cornerstone of hormonal regulation, particularly concerning insulin, testosterone, and cortisol [289]. Exercise has been demonstrated to enhance insulin sensitivity, thereby increasing NO bioavailability, particularly following donation or physical exertion [290]. Moderate aerobic activities, such as walking, cycling, or swimming, have been demonstrated to enhance vascular health while concomitantly reducing stress and regulating cortisol levels [291]. Chronic stress, characterised by elevated cortisol levels and impaired NO synthesis, can be mitigated through relaxation techniques such as meditation, yoga, or mindfulness [292]. Furthermore, resistance training has been demonstrated to elevate testosterone levels in both males and females, thereby promoting vascular health [293].

Sleep quality is also a key factor in regulating hormones, e.g., insulin, testosterone, and cortisol, particularly in blood donors [294]. Emerging evidence links disrupted sleep patterns to elevated cortisol levels and impaired insulin sensitivity, both of which adversely affect NO bioavailability [295]. Ensuring a consistent sleep schedule with 7–9 h of restful sleep each night is therefore essential for maintaining hormonal balance and vascular health [296]. Good sleep hygiene practices, such as avoiding caffeine and electronic devices before bedtime, have been shown to improve sleep quality, thereby enhancing NO production and vascular function [297].

A comprehensive approach that combines a nutrient-rich diet, regular physical activity, and healthy sleep patterns constitutes an effective strategy for improving hormonal balance, optimising NO production, and supporting vascular health. The integration of these lifestyle practices, in conjunction with the consumption of antioxidant-rich foods and spices, provides a comprehensive framework for the management of hormone-related dysfunction and the promotion of overall well-being [298].

Future research should focus on demographic variations in response to L-arginine, with particular emphasis on age- and sex-specific factors that influence its role in NO metabolism and vascular recovery. The investigation of the interaction between genetic predisposition and lifestyle factors will provide further insights into personalised interventions. Furthermore, the development of targeted strategies, such as the combination of L-arginine supplementation with tailored dietary plans, holds promise for improving donor recovery and preserving endothelial health. It is also crucial to assess the cumulative effects of repeated blood donation, particularly the long-term impact of oxidative stress on mitochondrial and vascular function. The development of strategies that minimise risks and enhance the well-being of frequent blood donors will be greatly informed by these efforts.

## 3. Conclusions

This article emphasises the pivotal role of L-arginine and nitric oxide in supporting recovery after blood donation. By exploring the molecular and physiological mechanisms involved, it underscores how NO enhances endothelial function, promotes vascular elasticity, and mitigates oxidative damage—key factors for the health and recovery of blood donors. The discussion on NO production in the context of repeated donation provides valuable insights into potential dietary and supplementation strategies aimed at optimising donor recovery.

Additionally, the maintenance of a balanced hormonal profile is increasingly being recognised as critical for preserving endothelial function. Disruptions in these pathways, whether resulting from hormonal imbalances or underlying health conditions, can lead to impaired vascular adaptation and heightened cardiovascular risk. This understanding guides strategies to safeguard donor health and carries broader implications for the sustainability and effectiveness of blood donation programmes.
